# Epithelial cells in nipple aspirate fluid and subsequent breast cancer risk: A historic prospective study

**DOI:** 10.1186/1471-2407-8-75

**Published:** 2008-03-19

**Authors:** Kimberly A Baltzell, Michelle Moghadassi, Terri Rice, Jennette D Sison, Margaret Wrensch

**Affiliations:** 1 University of California San Francisco, Department of Physiological Nursing, San Francisco, CA, USA

## Abstract

**Background:**

Past studies have shown that women with abnormal cytology or epithelial cells in nipple aspirate fluid (NAF) have an increased relative risk (RR) of breast cancer when compared to women from whom NAF was attempted but not obtained (non-yielders). This study analyzed NAF results from a group of women seen in a breast clinic between 1970–1991 (N = 2480). Our analysis presented here is an aggregate of two sub-groups: women with questionnaire data (n = 712) and those with NAF visits beginning in 1988 (n = 238), the year in which cancer case information was uniformly collected in California.

**Methods:**

Cytological classification was determined for a group of 946 women using the most abnormal epithelial cytology observed in fluid specimens. Breast cancer incidence and mortality status was determined through June 2006 using data from the California Cancer Registry, California Vital Statistics and self-report. We estimated odd ratios (ORs) for breast cancer using logistic regression analysis, adjusting for age. We analyzed breast cancer risk related to abnormality of NAF cytology using non-yielders as the referent group and breast cancer risk related to the presence or absence of epithelial cells in NAF, using non-yielders/fluid without epithelial cells as the referent group.

**Results:**

Overall, 10% (93) of the 946 women developed breast cancer during the follow-up period. Age-adjusted ORs and 95% confidence intervals (C.I.) compared to non-yielders were 1.4 (0.3 to 6.4), 1.7 (0.9 to 3.5), and 2.0 (1.1 to 3.6) for women with fluid without epithelial cells, normal epithelial cells and hyperplasia/atypia, respectively. Comparing the presence or absence of epithelial cells in NAF, women with epithelial cells present in NAF were more likely to develop breast cancer than non-yielders or women with fluid without epithelial cells (RR = 1.9, 1.2 to 3.1).

**Conclusion:**

These results support previous findings that 1) women with abnormal epithelial cells in NAF have an increased risk of breast cancer when compared to non-yielders or women with normal epithelial cells in NAF and 2) women with epithelial cells present in NAF have an increased risk of breast cancer when compared to non-yielders or women who had NAF without epithelial cells present.

## Background

Breast cancer is the second leading cause of cancer death in women in the United States [1]. Of the 180,000 women who will be diagnosed with breast cancer this year, approximately 40,000 will die of this disease. Determining who is at risk for breast cancer has proven to be an inexact science. Risk biomarkers with a lifetime positive predictive value for breast cancer of >30% are limited to individuals with a deleterious mutation in genes responsible for hereditary breast cancer or a diagnosis of lobular carcinoma in situ (LCIS) or atypical hyperplasia plus family history of breast cancer in a woman undergoing a diagnostic biopsy. The vast majority of women interested in risk assessment would not qualify for germline genetic testing nor have they undergone a diagnostic biopsy [2].

Since 95% of breast tumors arise in the lining of the milk ducts, evaluation of these ducts might be a means of identifying abnormal cells that could progress to cancer. Past studies have shown that women with abnormal cytology in nipple aspirate fluid (NAF) have an increased relative risk (RR) of breast cancer when compared to women with normal cytology in NAF and non-yielders (women from whom NAF was attempted but not obtained) [3,4]. In addition, a recent study found that women with epithelial cells present in NAF, regardless of cytological category, were more likely to develop breast cancer than women with NAF that did not contain epithelial cells or non-yielders [5].

This present study analyzed NAF results from a group of women seen by Dr. Otto Sartorius in his Santa Barbara breast clinic between 1970–1991. The purpose of this paper is to present the results of a historic prospective study determining 1) whether or not the type of epithelial cells present in NAF (normal, hyperplasia or atypia) influenced subsequent breast cancer development and 2) whether or not there was an increased risk of breast cancer development if epithelial cells were present in NAF, regardless of cytological category.

## Methods

### Study population

Subjects were a cohort of 3,203 women seen by Dr. Otto Sartorius in his Santa Barbara breast clinic between 1970–1991. The women were self-referred or referred by physicians. Dr. Sartorius performed all NAF collection during this period of time and utilized one pathologist to determine cytological diagnoses for all specimens. NAF collection/classification information and covariate information (specifically age) used in this analysis was abstracted from the subjects medical records by a team of registered nurses in Santa Barbara, California. Age was the only covariate information that could be consistently abstracted from the medical records. The Committee on Human Research of the University of California, San Francisco, and the Department of Defense (DOD) Human Subjects Research Review Board approved this study of human subjects.

### Inclusion/exclusion criteria

All women seen by Dr. Sartorius between 1970–1991 were eligible for inclusion in the study (N = 3,203). Women who were diagnosed with breast cancer at initial visit with Dr. Sartorius or within six months of initial visit with Dr. Sartorius were excluded from the study. Women were also excluded who did not have NAF collection attempted by Dr. Sartorius.

### Follow-up protocol

Beginning in October 2005, all women were mailed structured questionnaires and consent forms requesting information on personal and family history of breast cancer and reproductive factors associated with an increased breast cancer risk. After two mailings and no response we attempted to contact the women by telephone. Several methods were used to update the addresses of the non-responders, including requests to the California Department of Motor Vehicles (DMV), California Vital Statistics, and several internet search engines. A modified proxy questionnaire was sent to next-of-kin for subjects identified as deceased. Another source of information about breast cancer status was the California Cancer Registry (CCR), which did not uniformly begin collecting data on breast cancer cases in California until 1988. Follow-up ended in June, 2006.

The final model (N = 946) is composed of women who were seen by Dr. Sartorius between 1970–1991 (Table 1). Breast cancer status upon follow-up was determined either by a returned questionnaire or by linkage with the CCR. A comparison of the group of women included in the final analysis versus the women excluded from the final analysis is shown in Table 2.

**Table 1:** Population of eligible women used in final analysis, Sartorius Cohort 1970–1991.

	**3203**	Total number in tracking database
	(- 159)	No cytology results at all
	(- 200)	Results from histology only, no NAF attempted
	(- 364)	With breast cancer within 6-months of most severe NAF result
**TOTAL # ELIGIBLE**	**2480**	With NAF results and no breast cancer within 6-months of severe NAF result visit (note: this includes 9 women with breast cancer dates unknown)
		*Subset of women used in this analysis:*
	712	Women with questionnaire data
	238	Women seen 1/01/1988 or after
	950	
	(- 3)	"Unable to classify" NAF result
	(- 1)	Age missing (NAF date missing)
**Total number in model**	**946**	
**Total number not in model**	**1534**	

**Table 2:** Comparison between women excluded from the final analysis versus those included in the final analysis, Sartorius Cohort 1970–1991.

	**Excluded from Analysis**	**Included in Analysis**	
**Basic Stats**	**N = 1534**	**N = 946**	**ANOVA**
**Age at NAF visit:**			
Mean	43.9 *	42.6	
Median	42	41	
Std Err	0.39	0.42	
Min-max	16–89	16–87	
			p = 0.04
			
**Age at breast cancer diagnosis:**			
N	81 **	93	
Mean	62.0	60.8	
Median	62	62	
Std Err	1.5	1.27	
Min-max	30–89	34–86	
			p = 0.54
			
**Breast cancer:**	**n (%)**	**n (%)**	
	90 (6%)	93 (10%)	**Chi Square**
			*p *<*0.001*
			
**NAF Cytology:**	**n (%)**	**n (%)**	
No fluid	929 (61%)	714 (75%)	
Insufficient specimen	45 (3%)	19 (2%)	
Normal	232 (15%)	89 (9%)	
Hyperplasia	234 (15%)	97 (10%)	
Atypia	81 (5%)	27 (3%)	
[Unable to classify]	13 (1%)		
			*p *<*0.001*

* N = 1525

** 9 missing NAF age

### Nipple aspiration technique and cytologic classification

Nipple aspirate was obtained using the technique developed by Dr. Sartorius and has been described elsewhere [6,7]. The clinical database used for this study contained a variety of cytologic classifications. These classifications were condensed and categorized as either 1) normal cells 2) hyperplasia or 3) atypical hyperplasia by our study pathologist, Dr. Eileen King. [8]. For this analysis, the most abnormal epithelial changes observed in the fluid specimens were used for the date of age at study entry.

### Data analysis

This paper evaluates breast cancer risk in relation to NAF cytology results which were categorized as follows: no fluid obtained, fluid without epithelial cells, normal/benign, hyperplasia, atypia, and unable to classify. For women with multiple NAF results, the most abnormal result was used. While data on timing of cancer and death was available, precise dates on censoring of unaffected and living people was not and thus logistic regression was determined to be the appropriate analytic method for use in this study.

Two logistic regression models were used in the analyses presented here. The first model used the non-yielders group of women as the referent and calculated the odds ratio for women who had fluid without epithelial cells, normal, or hyperplasia/atypia combined. The decision to use the non-yielders as the referent is consistent with past studies and the hypothesis that women without NAF secretions are at a lower risk of developing breast cancer than women who produce breast fluids [3]. The second model tested the hypothesis that the presence of epithelial cells may be a risk factor for breast cancer. In this case the non-yielders and fluid without epithelial cells groups were combined as the referent. All other women comprised the non-referent group (normal, hyperplasia, atypia, and unable to classify). Both models adjusted for age at the time of the NAF visit.

The PROC LOGISTIC procedure in SAS version 9.1 [9] was used to generate the odds ratios for these analyses.

## Results

### Description of the cohort and follow-up

Of the 946 women in the final model, data on breast cancer status was obtained on 712 (75%) by completed questionnaire data and on 238 (25%) from linkage with the CCR database. The women with completed follow-up were between ages 16–87 at study entry, with a mean age of 42.6. Mean follow-up time was 20.7 years, the median was 20 years and the range was 0.10 to 35 years. The mean age at breast cancer diagnosis was 60.8 years with a range of 34–86. Seventy-five percent (714) of the cohort were non-yielders versus 24% (232) of women who produced fluid either with or without epithelial cells. As of June 2006, 93 of 946 (10%) women had developed breast cancer since enrollment in the study (Table 2). Of the breast cancer cases reported, 75 were self-reported and the remaining 18 were determined solely via the CCR.

### Breast cancer incidence by cytologic diagnosis

In our review of the type of epithelial cells present in NAF and subsequent breast cancer development, a progressive increase in breast cancer cases was seen with each progressive category of cytology (Table 3). Breast cancer incidence was 9% (63 of 714) in the non-yielders group, 11% (2 of 19) in women who had fluid without epithelial cells, 12% (11 of 89) in women with normal epithelial cells, and 14% (17 of 124) in women with hyperplasia/atypia. Women who had fluid without epithelial cells were 1.4 times more likely to develop breast cancer than non-yielders (95% CI, 0.3–6.4). Women with normal epithelial cells in NAF were 1.7 times more likely to develop breast cancer than non-yielders (95% CI, 0.9–3.5). Women with proliferative epithelial cells in NAF (hyperplasia/atypia) were 2 times more likely to develop breast cancer than non-yielders (95% CI, 1.1–3.6, p value = 0.02).

**Table 3:** Odds ratios of breast cancer risk by cytological categories and presence of epithelial cells in NAF, Sartorius Cohort 1970–1991.

**Breast cancer by cytology:**	**In model: N = 946 # with breast cancer**	**n**	**%**	**OR**	**95% CI**	**p-value**
No fluid	63	714	9%	1.0	(referent)	
Fluid without epithelial cells	2	19	11%	1.4	0.32–6.4	0.64
Normal	11	89	12%	1.7	0.87–3.5	0.12
Hyperplasia or atypia	17	124	14%	2.0	1.1–3.6	0.02
						
**Any epithelial cells:**						
No fluid/insufficient specimen	65	733	9%	1.0	(referent)	
Unable to classify, normal, hyperplasia, atypia	28	213	13%	1.9	1.2–3.1	0.01

### Breast cancer incidence by presence or absence of epithelial cells

An increased risk of breast cancer was also found in our comparison of solely the presence of epithelial cells in NAF, regardless of cytological category (Table 3). Women with any epithelial cells in NAF were 1.9 times more likely to develop breast cancer than non-yielders or women who had fluid from NAF that did not contain epithelial cells (95% CI, 1.2–3.1, p value = 0.01).

## Discussion

Interrogating the breast epithelium directly with core needle biopsy is an unrealistic tool for determination of risk for the majority of women without significant family history or other major risk factors for breast cancer. Nipple aspiration is an inexpensive, non-invasive method for evaluating breast epithelium. Tice et al. [10] found that NAF cytology may improve the Gail model's predictive ability, especially for women with high risk. Information regarding proliferative breast epithelium is currently obtained for use in the Gail model via biopsy, an invasive and unrealistic screening tool for large populations of women. Breast biopsies are performed once a woman is symptomatic, limiting their ability to provide predictive value in determining who is at risk for breast cancer. The use of NAF is a relatively inexpensive, non-invasive route for evaluating breast epithelium, the site of 90% of diagnosed breast cancer cases.

This study used the results from the NAF sample with the most abnormal results in the final analysis. In order to address the issue of possible bias introduced by using these subsequent samplings for some women, we re-ran our analysis using the data from the first sampling for all subjects. Our results were almost identical; all ORs and statistics remained the same. In addition, we re-analyzed the data on subjects stratified according to three age groups; less than 45 years, 45–54 years and 55 years or older. Our population in the less than 45 year old group was too small to analyze (N-48) and in the 45–54 year old age group, the number with breast cancer was only 6. Despite these small numbers, the trend was nearly identical to the original analysis combining all groups.

Our findings are consistent with the findings of other recent studies and appear to confirm that women with cellular NAF have a higher risk of cancer than those with no NAF or NAF without epithelial cells [3-5]. Wrensch et al. [4] found a 2–5 fold greater risk of breast cancer development in women with proliferative epithelial cells in NAF when compared to women with normal epithelial cells in NAF and non-yielders. Buehring et al. [5] found women with any epithelial cells in NAF had a 2-fold greater risk of breast cancer development when compared to non-yielders or women with NAF fluid that did not contain epithelial cells. These findings confirm that women with epithelial cells in NAF, normal or abnormal, are at a greater risk of developing breast cancer. Past studies have shown that younger women are more likely to yield NAF fluid for evaluation [7,11]. These women are less likely to benefit from current breast cancer screening modalities, making NAF an important adjunct to presently employed screening modalities.

There were limitations involved in the conducting of this study. First, due to the inability to accurately track breast cancer cases prior to 1988, we were unable to use information from the cohort without questionnaire data prior to 1988. In addition, the women in the study were visiting a breast clinic and the majority presented with breast symptoms for evaluation. Although no formal evaluation of breast cancer risk (i.e. the Gail model) was utilized by Dr. Sartorius, this cohort is likely to represent a high risk group and the results cannot be generalized to the population at large. Finally, the ethnic make-up of this cohort was predominantly white; therefore the relative risks found may not be applicable to other ethnicities with varying physiologic factors influencing breast epithelium.

## Conclusion

These results support our hypothesis that 1) women with abnormal epithelial cells in NAF have an increased risk of breast cancer when compared to non-yielders and women with normal epithelial cells in NAF and 2) women with epithelial cells present in NAF have an increased risk of breast cancer when compared to non-yielders and women without epithelial cells present in NAF. As early detection of breast cancer is critical for a cure, being able to identify women at high risk for breast cancer would justify closer follow-up and the use of multiple methods to ensure early detection. Examining NAF may enhance current risk prediction models and provide an easy and inexpensive way to help identify individuals at increased risk for breast cancer. Future studies are necessary to explain the association between our findings and increased breast cancer risk. It is necessary to test breast fluids for specific biomarkers in order to further the research on etiologic factors involved in breast carcinogenesis and true breast cancer prevention.

## Abbreviations

CCR = California Cancer Registry; CI = Confidence interval; DMV = Department of Motor Vehicles; LCIS = Lobular carcinoma in situ; NAF = nipple aspirate fluid; OR = odds ratio; PI = principal investigator; RR = relative risk.

## Authors' contributions

KB conceived of the study and set up the design and coordination of the research team. She also reviewed and interpreted all statistical analyses and drafted the original manuscript. MM performed all statistical analyses. TR and JDS participated in the design of the study and also performed statistical analyses. MW participated in the study design and coordination and helped to draft the manuscript. All authors read and approved the final manuscript.

## Pre-publication history

The pre-publication history for this paper can be accessed here:


